# The neuropsychiatric aftermath of exposure to weapons of mass destruction: applying historical lessons to protect health during the war in Ukraine

**DOI:** 10.1017/S0033291722002872

**Published:** 2023-08

**Authors:** Joshua C. Morganstein, Evelyn J. Bromet, Jun Shigemura

**Affiliations:** 1 Department of Psychiatry, School of Medicine, Uniformed Services University, 4301 Jones Bridge Road, Bldg B, 4th Floor, Bethesda, MD 20814; 2 Department of Psychiatry, Renaissance School of Medicine, Stony Brook University, 101 Nicolls Road, Health Sciences Center, Level 4, Stony Brook, NY 11794-8434; 3 Department of Health Sciences, Mejiro University, 320 Ukiya, Iwatsuki-ku, Saitama-shi, Saitama 339-8501

**Keywords:** Health anxiety, mental health and psychosocial services, posttraumatic stress disorder, war in Ukraine, weapons of mass destruction

The invasion of Ukraine (24 February 2022) created the greatest humanitarian disaster in decades. Millions have fled indiscriminate and targeted Russian bombardments, while the Ukrainian army and civilian territorial defense volunteers fight to protect Ukraine's sovereignty. There is added fear of radiation exposure at the Chornobyl (Chernobyl) and Zaporizhzhia nuclear power plants (NPPs) and the Kharkiv Physics and Technology Institute, commandeered by the Russian army. The NPP workers at Chornobyl were forced to continue operating the NPP for months at gun point by the Russian military, raising concerns about physical and mental fatigue among workers and the potential for radiation exposure from human and/or mechanical error beyond the confines of the nuclear facilities. Russian forces used the Zaporizhzhia plant to launch attacks at Ukraine with explosions occurring at the plant itself, prompting increased international concerns for a nuclear event. Compounding the unspeakable trauma of the war in Ukraine, the population faces threats of nuclear, as well as chemical and biological warfare from weapons of mass destruction (WMDs).

As of this writing, amidst the backdrop of the WMD threat, the destruction and atrocities at Marriupol and Buccha shocked the world with intensified shelling and fighting in the eastern and southern regions. Healthcare professionals within and outside Ukraine, including the Ukrainian Psychiatric Association, are providing care, training, and support to local mental health practitioners and directly to traumatized populations. Our research on Chornobyl and Fukushima have shown that general practitioners and other medical specialists will also need training to recognize and treat the many psychological scars of this war among children and adults of all ages (Havenaar, Bromet, & Gluzman, 2016; Shigemura et al., 2021).

## Mental health impact of war in Ukraine

The neuropsychiatric impact on survivors of war is multi-layered. The Ukrainian population are exposed to ongoing life-threatening attacks, witnessing death of friends and loved ones, torture and sexual violence, separation from family members, and destruction of homes and communities. Additionally, their displacement and readjustment to new environments creates major public mental health needs among evacuees and their new host countries. Though the resilience and altruism we are witnessing is exceptional, there will be a full range of mental health consequences including grief, anxiety, depression, sleep disorders, somatization, and post-traumatic stress disorder. Beyond war-related syndromes, we need to recognize the psychological and behavioral impacts of the use, and even threatened use, of WMD, and historical events offer important lessons learned.

## Psychological and behavioral impacts of WMD events: lessons from Japan

Japan was the site of the atomic bombings of Hiroshima and Nagasaki in World War II in 1945, sarin gas attacks in Matsumoto and Tokyo in 1994 and 1995, respectively, by the cult group Aum Shinrikyo, and one of the world's worst known NPP explosions during the triple disaster in Fukushima Prefecture in 2011. The recent global COVID-19 pandemic revealed that anxiety levels have been increased in otherwise healthy people, with those with mental health disorders, prior exposure to trauma, first responders, and healthcare workers being particularly susceptible (Shigemura & Kurosawa, 2020). Health studies of these events give us a wealth of knowledge in understanding the substantial and enduring effect of a WMD event.

A unique issue is that precise exposure during these environmental adversities is difficult to observe or quantify. In addition, distrust of agencies responsible for public health contributed to the widespread belief that one was exposed. That personal perception is an essential factor in determining psychological and behavioral response and once embraced, becomes enduring and leads to a range of mental ill-health conditions. A study of Nagasaki survivors found anxiety and somatization persisted more than 50 years regardless of actual radiation exposure (Kim et al., 2011). Atomic bomb survivors were exposed to widespread fear, discrimination, and stigmatization from the general population, exacerbating their worries about health and longevity. In turn, they experienced a decline in well-being and fear about future health effects for themselves and their families (e.g. cancer and transgenerational genetic effects) (Shigemura et al., 2017).

After the Tokyo subway sarin attacks, large numbers of unexposed people rushed to receive medical care, overwhelming local healthcare facilities. Among the injured, physical and ophthalmic symptoms became persistent and were associated with posttraumatic stress symptoms two decades after the attack (Nakamine, Kobayashi, Fujita, Takahashi, & Matsui, 2018). Just like in Chornobyl, mothers of young children and nuclear plant workers were particularly affected in Fukushima (Havenaar et al., 2016; Shigemura et al., 2021). In Fukushima, rates for adverse mental health remained high, even 8 years post-disaster (nonspecific psychological distress, 8.3–65.1%; depressive symptoms, 12–52.0%; and posttraumatic stress symptoms, 10.5–62.6%) (Shigemura et al., 2021). Medical tests to evaluate radiation effects were associated with both worsening and mitigating anxiety and subjective well-being, underscoring the importance of appropriate health risk communications between healthcare workers and affected communities (Murakami et al., 2018).

## How should healthcare professions prepare for WMD events?

When WMD events take place, it is often unclear when one has been exposed. Regardless of actual exposure, this uncertainty leads to increased distress and surges in healthcare demand. When people present for healthcare, it is frequently to emergency and primary care with concerns for physical symptoms. To address lowered health perceptions, somatization, and medical-psychiatric comorbidities, it will be essential to enhance collaborations between physical and mental health services through increased communication, education, and embedding mental health personnel within these other services (Gouweloos, Dückers, te Brake, Kleber, & Drogendijk, 2014). Since the Fukushima disaster, the World Health Organization and other organizations developed mental health and psychosocial support guidelines to address post-nuclear disaster challenges (World Health Organization, 2020). In addition, Psychological First Aid offers practical interventions to protect community mental health through actions that enhance the five essential elements of safety, calming, social connectedness, efficacy, and hope (Hobfoll et al., 2007). Conceivably these and other resources provided by the Red Cross, the International Medical Corps, and non-governmental organizations might serve as backbones for WMD disaster preparedness strategies.

Previous WMD disasters revealed that assessment of risk and protective factors before, during, and after the event are crucial in providing optimal care for affected people (Shigemura et al., 2017). These factors include, but are not limited to, prior and current mental health and trauma exposure, physical health conditions, socioeconomic conditions, social support, risk and health perceptions, and stigma. It is important for healthcare workers to respect and acknowledge patient concerns about exposure to invisible WMD agents while also providing timely medical evaluation. Healthcare workers can build trust and enhance protective health behaviors within communities through empathic listening as well as clear and understandable messages. It is also crucial that the compound effects of war and WMD trauma be anticipated and evaluated. For instance, victims of physical or sexual violence in war may have enhanced fear response to perceived WMD exposure. In humanitarian or emergency settings, utilization of brief, simple fact sheets with specific actions to protect health might be useful to enhance communication with patients who are under extreme stress and may be unable to assimilate complex health information (Center for the Study of Traumatic Stress, 2022). It is important for healthcare workers to remember that while these exposures might have occurred suddenly and quickly, the care many people will need could be very long-lasting.

Healthcare workers also experience unique challenges during WMD events likely to be complicated by stress on global healthcare systems from the COVID-19 pandemic, which itself offers lessons learned to protect the health of workers (Morganstein & Flynn, 2021). Fear for personal and family safety from exposure or contamination following work activities are common, and best addressed through ensuring adequate supplies and training in personal protective equipment use and procedures, such as decontamination. Organizational factors within healthcare systems, such as timely, updated crisis communication messaging, as well as clear and consistently applied policies and procedures reduce distress and enhance functioning for healthcare workers (Brooks, Dunn, Amlôt, Rubin, & Greenberg, 2018). Peer support during the stress of WMD events can foster a sense of safety and connectedness to bridge isolation that results from ongoing uncertainty and required use of protective barrier equipment. Leadership actions, such as role modeling of self-help and stress management, as well as being present with personnel in their workspace to address concerns and remind them of the value of their work are actions that protect health and enhance operational sustainment (Geerts et al., 2021).

## Conclusions

The evolving war in Ukraine raises global concerns about the potential for future WMD events and their compounding traumatic effect on the Ukraine within and outside its borders. Whether or not a significant WMD event occurs, the invisible and imperceptible nature of these events add considerable fear and uncertainty to the numerous traumatic exposures experienced by the affected communities. Healthcare workers will need to understand psychosocial consequences of WMD, on patients and themselves, and prepare accordingly. It is also crucial to keep in mind that people have the power to recover even through the hardest of times, as shown in the recovery of Japanese communities after the atomic bombings, Jews and other victims of the Holocaust, prisoners of war, populations affected by the Fukushima disaster, and survivors of neurotoxic exposures.

## References

[ref1] Brooks, S. K., Dunn, R., Amlôt, R., Rubin, G. J., & Greenberg, N. (2018). A systematic, thematic review of social and occupational factors associated with psychological outcomes in healthcare employees during an infectious disease outbreak. Journal of Occupational and Environmental Medicine, 60(3), 248–257. doi: 10.1097/jom.0000000000001235 29252922

[ref2] Center for the Study of Traumatic Stress, Department of Psychiatry, Uniformed Services University of the Health Sciences, Bethesda. (2022). War in Ukraine Mental Health Resources. Retrieved from https://www.cstsonline.org/resources/resource-master-list/war-in-ukraine-mental-health-resources.

[ref3] Geerts, J. M., Kinnair, D., Taheri, P., Abraham, A., Ahn, J., Atun, R., … Bilodeau, M. (2021). Guidance for health care leaders during the recovery stage of the COVID-19 pandemic: A consensus statement. JAMA Network Open, 4(7), e2120295–e2120295. doi: 10.1001/jamanetworkopen.2021.20295 34236416

[ref4] Gouweloos, J., Dückers, M., te Brake, H., Kleber, R., & Drogendijk, A. (2014). Psychosocial care to affected citizens and communities in case of CBRN incidents: A systematic review. Environment International, 72, 46–65. 10.1016/j.envint.2014.02.009. 24684819

[ref5] Havenaar, J. M., Bromet, E. J., & Gluzman, S. (2016). The 30-year mental health legacy of the Chernobyl disaster. World Psychiatry, 15(2), 181–182. doi: 10.1002/wps.20335 27265712 PMC4911770

[ref6] Hobfoll, S. E., Watson, P., Bell, C. C., Bryant, R. A., Brymer, M. J., Friedman, M. J., … Ursano, R. J. (2007). Five essential elements of immediate and mid-term mass trauma intervention: Empirical evidence. Psychiatry, 70(4), 283–315. doi: 10.1521/psyc.2007.70.4.283 18181708

[ref7] Kim, Y., Tsutsumi, A., Izutsu, T., Kawamura, N., Miyazaki, T., & Kikkawa, T. (2011). Persistent distress after psychological exposure to the Nagasaki atomic bomb explosion. British Journal of Psychiatry, 199(5), 411–416. doi: 10.1192/bjp.bp.110.085472 22045947

[ref8] Morganstein, J. C., & Flynn, B. W. (2021). Enhancing psychological sustainment & promoting resilience in healthcare workers during COVID-19 & beyond: Adapting crisis interventions from high-risk occupations. Journal of Occupational and Environmental Medicine, 63(6), 482–489. doi: 10.1097/jom.0000000000002184 33710105 PMC8168667

[ref9] Murakami, M., Takebayashi, Y., Takeda, Y., Sato, A., Igarashi, Y., Sano, K., … Tanigawa, K. (2018). Effect of radiological countermeasures on subjective well-being and radiation anxiety after the 2011 disaster: The Fukushima health management survey. International Journal of Environmental Research and Public Health, 15(1), E124. doi: 10.3390/ijerph15010124. PMC5800223 29329263

[ref10] Nakamine, S., Kobayashi, M., Fujita, H., Takahashi, S., & Matsui, Y. (2018). Posttraumatic stress symptoms in victims of the Tokyo subway sarin attack: Twenty years later. Journal of Social and Clinical Psychology, 37(10), 794–811. doi: 10.1521/jscp.2018.37.10.794

[ref11] Shigemura, J., Harada, N., Tanichi, M., Nagamine, M., Shimizu, K., & Yoshino, A. (2017). Nuclear disaster response. In R. J. Ursano, C. S. Fullerton, L. Weisaeth, & B. Raphael (Eds.), Textbook of disaster psychiatry (2nd ed., pp. 298–313). Cambridge: Cambridge University Press.

[ref12] Shigemura, J., & Kurosawa, M. (2020). Mental health impact of the COVID-19 pandemic in Japan. Psychological Trauma, 12(5), 478–479. doi: 10.1037/tra0000803 32525392

[ref13] Shigemura, J., Terayama, T., Kurosawa, M., Kobayashi, Y., Toda, H., Nagamine, M., & Yoshino, A. (2021). Mental health consequences for survivors of the 2011 Fukushima nuclear disaster: A systematic review. Part 1: Psychological consequences. CNS Spectrums, 26(1), 14–29. doi: 10.1017/s1092852920000164 32192553

[ref14] World Health Organization. (2020). A framework for mental health and psychosocial support in radiological and nuclear emergencies. Geneva. Retrieved from https://www.who.int/publications/i/item/9789240015456.

